# Operative and Pathological Factors in Right-Sided Colon Cancers: How Can We Improve the Outcomes?

**DOI:** 10.7759/cureus.33832

**Published:** 2023-01-16

**Authors:** Soraya F Conroy, Leigh R Biddlestone, Edward Courtney

**Affiliations:** 1 Department of Surgery, Royal United Hospital, Bath, GBR; 2 Department of Cellular Pathology, Royal United Hospital, Bath, GBR

**Keywords:** complete mesocolic excision, lymph node yield, mismatch repair status, extramural venous invasion, colon cancer

## Abstract

Introduction: Though the tumour-node-metastasis staging classification is the standard approach to risk stratification in patients with colorectal cancer, several other important variables including the presence of extramural venous invasion (EMVI), the tumour mismatch repair status, as well as surgical technique and its influence on lymph node yield all have an impact on long-term survival. This study aims to review both the impact of the type of operation on lymph node yield: complete mesocolic excision (CME) versus right hemicolectomy, and the impact of EMVI and microsatellite instability in predicting overall survival in patients undergoing a right hemicolectomy for colon cancer.

Methods: Data of all patients who underwent an elective or emergency right hemicolectomy with curative intent for colon cancer between January 2013 and June 2022 (inclusive) was collected for this single-centre retrospective study. Kaplan-Meier survival curves were calculated using the Statistical Package for the Social Sciences (SPSS version 28, IBM Corp., Armonk, NY) software, and the log-rank (Mantel-Cox) test was used to compare survival distribution between different groups.

Results: A total of 421 patients underwent a right hemicolectomy for colon cancer with curative intent during the study period. EMVI was present in 173 (41%) tumours. Survival analysis showed significantly reduced cancer-related survival in patients with EMVI-positive tumours (p < 0.001), with five-year survival rates of 70% in EMVI-positive groups versus 96% in EMVI-negative groups. Subgroup analysis showed a significant difference in survival between node-positive and node-negative tumours in cancers found to have EMVI (p < 0.001). Mean lymph node yield was significantly higher in the CME group versus the standard right hemicolectomy group (p < 0.001). We found no significant difference in survival between patients with microsatellite instability-high (MSI-H) tumours and microsatellite stable (MSS) tumours (p = 0.432).

Conclusion: Consideration of tumour biology and adopting the optimum surgical technique are factors that may influence long-term survival in patients with colorectal cancer. Extramural venous invasion is an important prognostic indicator of adverse outcomes in patients with right-sided colon cancer. Our study demonstrates a reduction in survival in patients with EMVI-positive tumours when undertaking subgroup analysis by the presence or absence of nodal disease. Further research needs to be undertaken to compare the relative efficacy of neoadjuvant versus adjuvant chemotherapy in right-sided cancers known to be EMVI-positive as some patients will fail to have adjuvant chemotherapy due to postoperative complications, thereby delaying recovery and missing the optimum window for treatment.

## Introduction

Colorectal cancer is a leading cause of cancer-related mortality worldwide and was the second most common cause of cancer deaths globally in 2020 [1]. Pathological staging not only forms the basis of prognostic estimations for patient survival but is essential to informing decision-making regarding ongoing oncological treatment following a colon cancer resection.

Tumour-node-metastasis (TNM) classification is a widely adopted approach used to predict the prognosis of patients with colon cancer and to guide adjuvant chemotherapy after surgery is undertaken. In addition to the TNM staging, extramural venous invasion (EMVI) has also been identified as an important prognostic indicator in patients with colon cancer [2-4]. As such, the Royal College of Pathologists includes vascular invasion, specifically venous, as one of the microscopic core data items to be included in histopathology reports for colorectal cancers [5]. Pathologists are required to audit venous invasion detection rates in resection specimens with a benchmark frequency of 30%, and it is recognised that the use of elastin stains can facilitate the detection of venous invasion in histological sections. Though EMVI does not directly affect the overall TNM staging of a tumour, its association with decreased long-term and disease-free survival highlights the importance of considering EMVI when planning further adjuvant treatments [3,4].

In addition to the detection of cancer cells within individual lymph nodes, the overall lymph node yield is also a key element in the pathological assessment of a resected colon cancer specimen. The relationship between increased lymph node yield and improved survival in patients with colon cancer has been reported in several studies [6,7]. Even in node-negative colon cancer, an increased lymph node yield has been associated with better survival outcomes [8]. The complete mesocolic excision (CME) approach, as first described by Hohenberger et al., in the resection of right-sided colon cancer has been shown, in some studies, to have superior outcomes when compared to conventional right hemicolectomies and has been associated with significantly higher lymph node yield [9,10].

Evidence suggests that right-sided colon cancers have worse outcomes than left-sided colon cancers [11,12]. Cancers arising from the right colon are more commonly mucinous tumours, are more likely to be associated with microsatellite instability, CpG island methylation, and BRAF mutations, and may be less responsive to neo-adjuvant chemotherapy and other treatments such as EGFR therapy [11-14]. Defects in MMR (mismatch repair) genes lead to microsatellite instability, which in turn accounts for approximately 15% of sporadic colorectal cancers. Identifying patients who have MMR gene-deficient or proficient tumours is another important aspect when considering tumour biology and the appropriateness of subsequent adjuvant therapies [15].

This study aims to assess the operative and pathological factors influencing cancer-related survival in patients with right-sided colon cancers. We aim to review both the impact of the type of operation on lymph node yield: CME versus right hemicolectomy, and the impact of EMVI and microsatellite instability in predicting overall survival in patients undergoing a right hemicolectomy for colon cancer.

Preliminary work related to this paper was presented at the American Society of Colon and Rectal Surgeons Annual Scientific Meeting in the "Future Leaders" podium presentation session on May 2nd, 2022.

## Materials and methods

Data for all patients who underwent an elective or emergency right hemicolectomy with curative intent for colon cancer between January 2013 and June 2022 (inclusive), were collected for this single-centre retrospective study. We included patients between January 2013 and June 2022 (inclusive) for analysis of lymph node yield in CME versus non-CME resections. All patients between January 2013 and December 2020 (inclusive) were included in EMVI survival analysis, and for MMR survival analysis, all patients between January 2017 and December 2020 (inclusive) were included. Follow-up for survival analysis was completed until July 2022.

Data regarding patient demographics, tumour characteristics, and operative technique were obtained from a prospectively maintained database. When missing data were identified, a retrospective data collection was undertaken using hospital electronic records where operation notes, pathology data, multidisciplinary team (MDT) records, oncology letters, and follow-up/surveillance information were stored. Patients for whom the operation was performed with palliative intent, those who died within 90 days of the operation, those who received neoadjuvant chemotherapy, or those who were found to have T1 cancer after pathology assessment were all excluded.

The operative technique was either right hemicolectomy or CME. For right hemicolectomies, the ileocolic, right colic (if present), and, in some cases, the right branch of the middle colic artery were taken. For CME operations, resections extended to include D3 lymph nodes and ligation at the origin of the appropriate colic vessels (i.e., central vascular ligation, CVL). When considering CVL, the ileocolic artery was always taken, and either the right branch of the middle colic artery (MCA) at the origin of its bifurcation from the main trunk of the MCA or, for tumours in the mid transverse colon, the MCA at its origin was taken.

All colon cancer resection specimens were reported using the guidelines set out in the UK Royal College of Pathologists dataset for histopathological reporting of colorectal cancer and reviewed at the time of MDT discussion by a pathologist with a special interest in gastrointestinal pathology [5]. MMR protein expression/microsatellite instability was assessed in all resected carcinomas following the publication of the 2017 National Institute for Health and Care Excellence (NICE) guidance on molecular testing for Lynch syndrome [16].

The presence of EMVI and MMR status with cancer-related survival were the primary outcomes. Kaplan-Meier survival curves, with estimated five-year survival probabilities, were calculated. Statistical analysis was performed using the Statistical Package for the Social Sciences (SPSS version 28, IBM Corp., Armonk, NY) software, and the log-rank (Mantel-Cox) test was used to compare the survival distribution between different groups. Patients’ electronic records, imaging, and MDT outcomes were used for follow-up.

## Results

**Figure 1:**
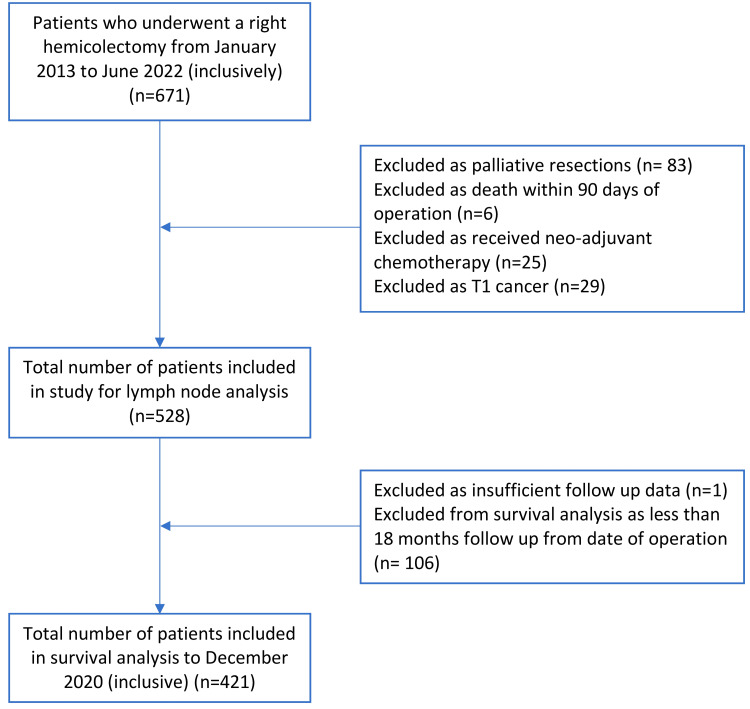
Patients included in the study

As illustrated in Figure 1, a total of 671 potential patients were identified for inclusion in this study. Eighty-three patients were excluded as the operation was not for curative intent, six were excluded as they died within 90 days of the operation, 25 were excluded as they had neoadjuvant chemotherapy, and 29 were excluded as they had T1 cancers. For survival analysis, 107 patients were excluded as they had less than 18 months follow-up from the date of operation or there was insufficient data on follow-up. Table 1 summarises patient and tumour characteristics.

**Table 1:** Characteristics of patients and tumours (n = 421)

Patient/Tumour Characteristics	n (%)
Female	216 (51%)
Male	205 (49%)
Age median/range (years)	74.8/23-92
Elective	354 (84%)
Emergency	67 (16%)
Extramural venous invasion positive	173 (41%)
Adjuvant chemotherapy	164 (39%)
Site	
Caecum	185 (44%)
Ascending colon	122 (29%)
Hepatic flexure	39 (9%)
Proximal transverse colon	28 (7%)
Mid transverse colon	31 (7%)
Distal transverse colon	16 (4%)
Access	
Laparoscopic	326 (77%)
Open	74 (18%)
Converted to open	21 (5%)
T-stage	
T2	48 (11%)
T3	253 (60%)
T4	120 (29%)
N-stage	
N0	260 (62%)
N1	112 (27%)
N2	49 (11%)
Lymph node yield median/range	23/2-69

**Figure 2:**
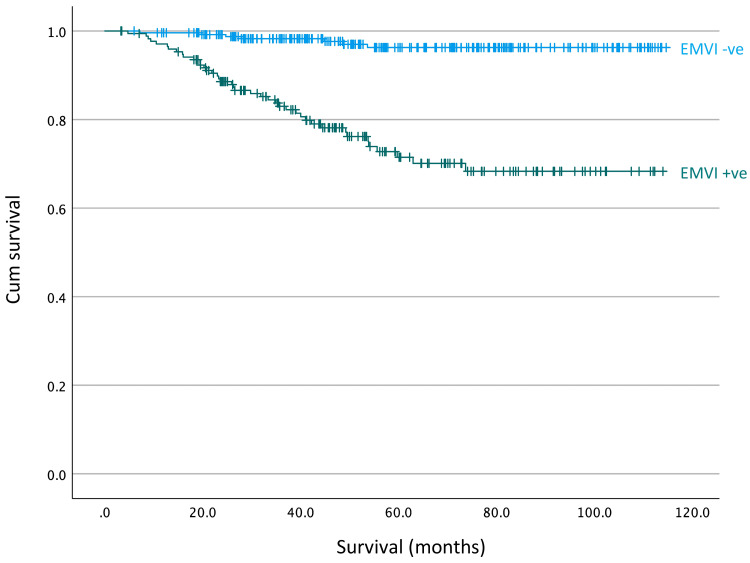
Survival curves for patients with and without EMVI, unadjusted for nodal stage (p < 0.001, log-rank analysis) EMVI: Extramural venous invasion.

With regards to EMVI status, 173 cancers were EMVI positive (41%). Survival analysis showed significantly reduced survival in patients with EMVI-positive tumours, unadjusted for nodal or tumour stage (p < 0.001), with five-year survival rates of 70% in EMVI-positive groups versus 96% in EMVI-negative groups (Figure 2).

**Figure 3:**
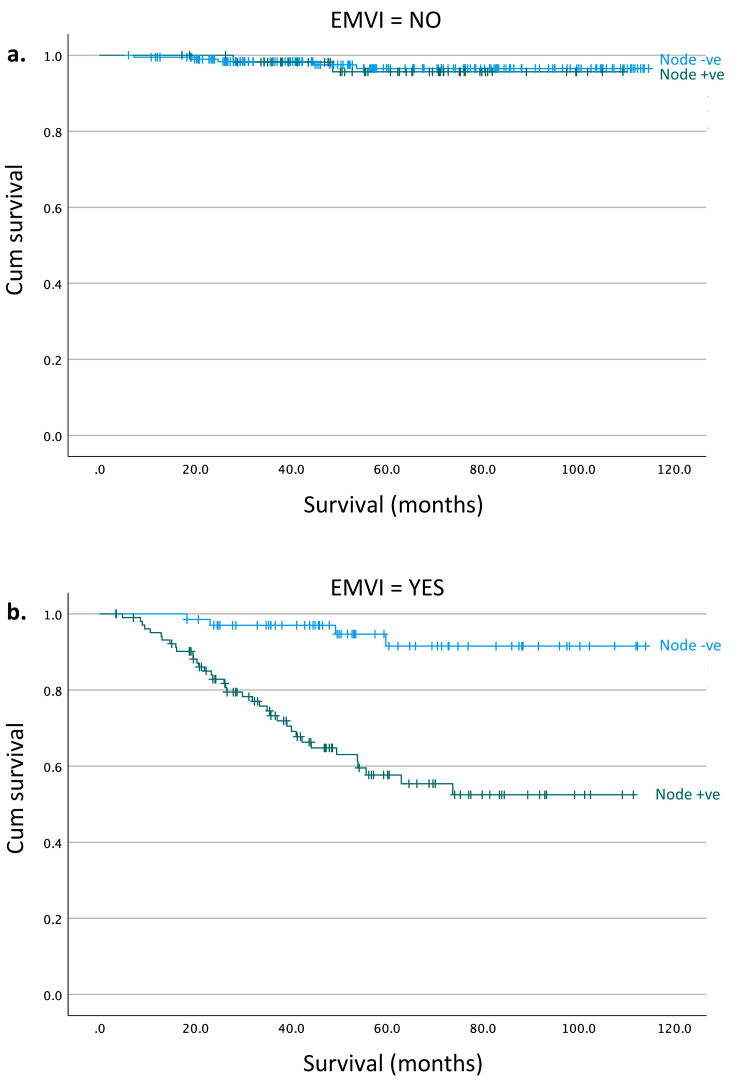
Survival curve of EMVI-negative tumours comparing tumours with negative versus positive lymph node involvement (p = 0.827, log-rank analysis). (b) Survival curve of EMVI-positive tumours comparing tumours with negative versus positive lymph node involvement (p < 0.001, log-rank analysis). EMVI: Extramural venous invasion.

When undertaking subgroup analysis of EMVI-negative tumours with nodal status, we found no significant difference between node-positive and node-negative tumours (p = 0.827, log-rank analysis) (Figure 3, Panel a). Further subgroup analysis found a significant difference in survival between node-positive and node-negative tumours in cancers that were also found to be EMVI-positive (Figure 3, Panel b) (p < 0.001, log-rank analysis). Five-year survival in the EMVI-positive and lymph node-positive group was 55% compared to just over 90% in the EMVI-positive and lymph node-negative group (Figure 3, Panel b).

**Figure 4:**
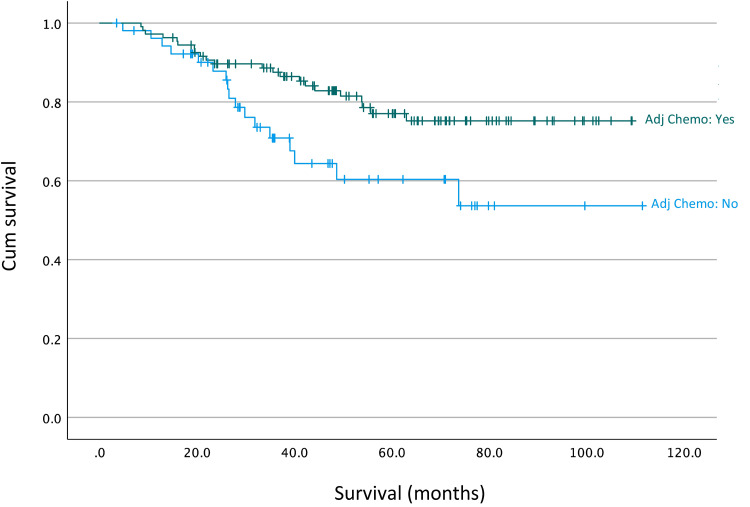
Survival curves for patients with node-positive disease comparing those who received adjuvant chemotherapy to those who did not (p = 0.018, log-rank analysis).

Of the 421 patients included in the survival analysis, 164 patients received adjuvant chemotherapy. When analysing the effects of adjuvant chemotherapy, we found significantly improved survival in patients with node-positive disease who received chemotherapy versus those who did not (p = 0.018) (Figure 4). There were a total of 56 EMVI-negative/lymph node-positive patients, and 41/56 (73%) of these patients received adjuvant chemotherapy.

**Figure 5:**
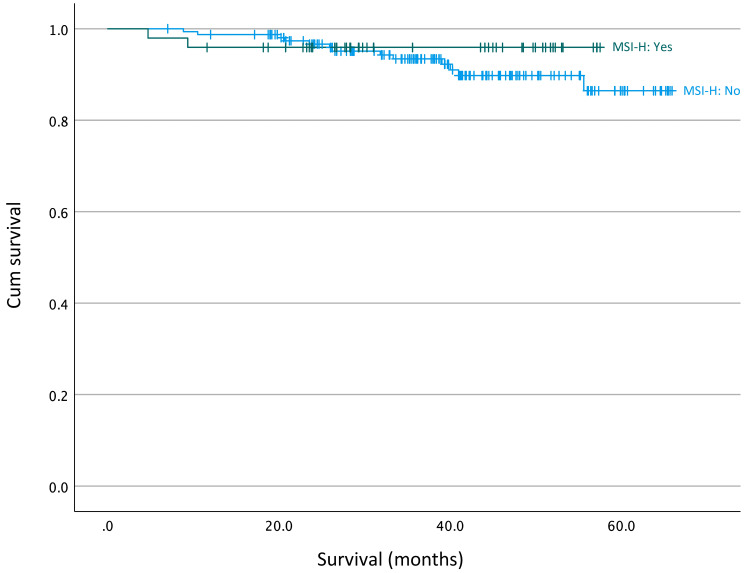
Survival curves for patients with microsatellite instability-high (MSI-H) tumours compared to those with microsatellite stable (MSS) tumours (p = 0.432, log-rank analysis)

Survival analysis by MSI status included a total of 206 patients from January 2017 to December 2020 (inclusive). Of these, 49 tumours were microsatellite instability-high (MSI-H) tumours. We found no significant difference in the survival of patients with MSI-H tumours compared to those with microsatellite stable (MSS) tumours (p = 0.432, log-rank analysis) (Figure 5).

**Figure 6:**
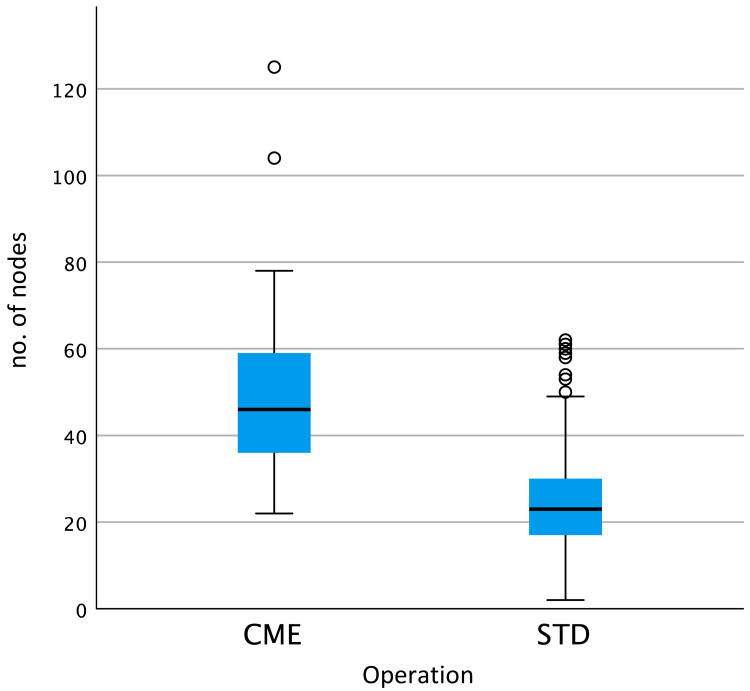
Lymph node yield by type of operation. The lymph node yield was significantly higher in the complete mesocolic excision group (mean: 52, median: 46) versus the standard right hemicolectomy group (mean: 25, median: 23) (p < 0.001, Kruskal-Wallis test). CME: Complete mesocolic excision right hemicolectomy; STD: Standard right hemicolectomy.

When comparing lymph node (LN) yield by the type of operation, we found that LN yield was significantly greater in the CME group (mean LN yield: 52) when compared with the standard right hemicolectomy group (mean LN yield: 25) (p < 0.001, Kruskal-Wallis test) (Figure 6).

## Discussion

To our knowledge, this is the first study comparing overall survival outcomes of patients with and without EMVI in those with specifically right sided colon cancer. Given that right-sided cancers are associated with worse survival outcomes when compared to left-sided colonic cancers, our study has several important implications when considering the clinical management of patients with right-sided cancer [11,12]. The results of this study have shown that the presence of EMVI in patients with right colon cancer confers a significantly reduced five-year survival rate when compared to tumours without EMVI. When undertaking subgroup analysis, a notable finding was the reduction in overall survival in lymph node-positive tumours that also had EMVI. This is consistent with other studies that showed EMVI as a poor prognostic indicator for survival, resulting in more aggressive disease [3,17]. When compared to lymph node disease, EMVI has a strong association with synchronous metastatic disease: with a study by Siddiqui et al. suggesting an almost four-fold risk of developing metastases after surgery in patients with EMVI-positive rectal cancer [18]. Tumours with EMVI have access to distant metastatic sites by means of the venous system, allowing more direct and rapid dissemination of cancer cells to distant sites when compared to the lymphatic system [19].

Adjuvant chemotherapy is not indicated in patients who have EMVI-negative/node-negative cancer. However, given the established importance of nodal involvement and long-term survival in patients with cancer, as reflected in the weight given to the nodal aspect of TNM staging, patients who have nodal involvement will often receive adjuvant chemotherapy. This is reflected in our study where 73% of patients who were EMVI-negative but node-positive received chemotherapy. This may explain the lack of impact of LN status in the EMVI-negative group, given that most lymph node-positive patients received chemotherapy. This is encouraging as it clearly shows a benefit with chemotherapy such that there is no difference in survival between the EMVI-negative/node-positive and the EMVI-negative/node-negative cohorts. The use of adjuvant chemotherapy is the main confounding bias within our study. If adjuvant chemotherapy was not used routinely, then we would be able to interpret the effect of pathological factors on outcomes more accurately.

In addition to a high propensity toward metastatic disease, the very presence of EMVI may be suggestive of more aggressive cancer. This may be in part due to epithelial-mesenchymal transition (EMT): a process in which cancer cells move freely and detach from other epithelial cells. For cancer cells to invade blood vessels, they must penetrate the basement membrane of a vessel, adhere to and penetrate the vascular epithelium, and then survive in the turbulent environment of the blood [20]. When compared to invasion of the lymphatic vessels, which offers little resistance to invading cancer cells due to their thin-walled low-pressure system, it could be hypothesised that cancers with EMVI have a more malignant potential as exhibited by their ability to invade these larger blood vessels. This further supports the need to accord more significance to EMVI in the staging of colon cancer.

Given the higher aggressive propensity of right-sided cancers and their association with inferior long-term outcomes, further efforts need to be made to effectively stage these cancers preoperatively to plan appropriate, tumour-specific, neoadjuvant treatment. The role of preoperative MRI in rectal cancers is well established and used to select patients with rectal cancer who would benefit from neoadjuvant chemoradiotherapy. In colon cancer, CT imaging is the modality used to stage tumours preoperatively. There is conflicting evidence regarding the suitability of preoperative CT for T staging. In a pilot study for the FOxTROT trial, Dighe et al. assessed the accuracy of CT in identifying patients with high-risk colon cancer and found that CT had 95% sensitivity and 50% specificity in identifying T3/T4 tumours and deemed CT a feasible tool to radiologically guide neoadjuvant chemotherapy for the trial [21]. Conversely, in a national population-based study of over 4,000 patients, Sjövall et al. concluded that preoperative CT did not result in accurate T staging [22]. Despite these differing opinions on the suitability of CT for T staging, there is a consensus that CT is unreliable in the detection of nodal involvement [21-23]. Fernandez et al. showed low sensitivity of 47% for preoperative CT detection of nodal disease. In addition to this, they showed an extremely low sensitivity of 8% for the detection of EMVI but high specificity of 91% [23].

Though CT imaging may be reliable for T staging, given its unreliability in N staging, it is difficult to conclude whether neoadjuvant chemotherapy would be suitable in patients with right-sided colon cancers. The preliminary results of two-year outcomes from the FOxTROT trial showed that patients who had pre- and postoperative chemotherapy versus those who had postoperative chemotherapy alone had a significantly reduced risk of recurrence (p = 0.042) (published in abstract form only) [14]. Only patients with high-risk cancer, as detected on preoperative CT imaging, with predicted T3-4 N0-2 were enrolled. Despite the overall promising results for neoadjuvant chemotherapy in these patients, when analysing the subgroups based on primary colonic tumour location, the benefit of neoadjuvant chemotherapy was only seen in left-sided colon cancers, with no significant reduction in recurrence in patients with right-sided colon cancer who received neoadjuvant chemotherapy (odds ratio: 0.97, 95% CI: 0.62-1.52) (published in abstract form only) [14]. Given the unreliability of CT in preoperative N staging, patients who were excluded from this study may have been suitable candidates for neoadjuvant treatment based on histopathological evaluation of their tumours. In addition, when considering the significant association of EMVI with inferior survival outcomes, the identification of EMVI preoperatively should be considered when selecting those for neoadjuvant chemotherapy. Given MRI is the standard preoperative imaging modality used in rectal cancer to guide neoadjuvant treatment, emerging evidence suggests that MRI has a role to play in staging patients with colon cancer [24]. Rollvén et al. reported greater detection by two separate observers of T-stage, N-stage, and EMVI when using MRI compared to CT imaging [24]. Though this study is limited by its small sample size and further studies are required to confirm the suitability of MRI in preoperative staging of right-sided colon cancer, it provides promising alternatives for more accurate detection of unfavourable pathological features to help guide neoadjuvant treatments.

The development of colon cancer is often multifactorial with several environmental and genetic factors leading to its development. Tumours with defective MMR genes show the presence of microsatellite instability, i.e., MSI-H which accounts for approximately 15% of sporadic colorectal cancers [25]. Patients with MSI-H tumours, i.e., MMR-deficient, have better survival when compared to those with MSS tumours, i.e., MMR-proficient. We did not find a significant difference between MSI-H tumours and MSS tumours (p = 0.432) most likely due to the small number of data points. Interestingly, MMR status has been shown to predict responsiveness to adjuvant treatments. Patients with MMR-deficient tumours (i.e., those with MSI-H) do not appear to derive benefit from 5-FU-based chemotherapy, but there is evidence to suggest that immune checkpoint inhibitors are beneficial in the neoadjuvant setting in non-metastatic colon cancer [26,27]. Understanding the MMR status of right-sided cancers is another key factor for the MDT to consider when deciding upon neoadjuvant treatments, given the higher propensity of right-sided cancers to be associated with high microsatellite instability when compared to left [28].

Identification of MSI-H tumours will highlight the patients who are less likely to benefit from neoadjuvant chemotherapy and should instead go straight to surgery, given that the morbidity associated with neoadjuvant chemotherapy will be less acceptable due to the lack of added benefit on survival in patients with these cancers [26]. Identification of MSI-H tumours preoperatively may also identify patients who may benefit from neoadjuvant immune checkpoint inhibitor therapy, adding a further argument to preoperative testing for MMR status [27]. In our centre, we routinely test all resected colon cancers for MSI-H and test preoperative biopsies if the oncologists are considering neoadjuvant treatment. Though there is no association between the presence of EMVI and MMR-deficient tumours, if improvements are made in preoperative detection of other high-risk features such as EMVI, this may increase the use of neoadjuvant therapies and would be further justification for testing all preoperative biopsies for MSI-H [29].

In addition to the differing biology of right- versus left-sided cancers, the surgical approach to resection is more variable in right-sided cancers, and this may in part contribute to the worse prognosis seen in these patients [11]. Since the widespread adoption of the surgical technique of total mesorectal excision (TME) when resecting rectal cancers, outcomes for patients with these cancers have dramatically improved [11,30]. As first described by Hohenberger et al. in 2009, CME is a radical approach to resection of right-sided colon cancers and has shown to lead to greater lymph node yield [9,10]. This is further supported by our study, showing significantly increased LN yield in the CME group (p < 0.001). Though some studies have shown that the CME approach for the resection of right-sided colon cancer may improve outcomes when compared to conventional right hemicolectomies, there is a lack of published data from randomised control trials to support the use of CME as the standard technique when resecting right-sided colon cancers [9,10]. In addition, the CME approach is a more technically demanding operation with higher risk of complications such as vascular injury and chyle leak and is therefore not the routinely used operative approach [31].

The strength of this study is the rigorous postoperative follow-up and surveillance, ensuring that the influence of pathological outcomes in this cohort can be accurately determined. However, as it is a single-centre study, it is limited by its modest sample size, limiting statistical power for certain analyses. Despite this, our results show significant findings consistent with a growing body of evidence in relation to EMVI and colorectal cancer.

## Conclusions

EMVI is an important independent prognostic indicator of adverse outcomes in patients with right-sided colon cancer. Our study demonstrates a significant reduction in survival in patients with EMVI-positive tumours when subclassified by the presence or absence of nodal disease. Pathological detection of EMVI should be a key factor for the MDT to consider when planning subsequent treatment. As CT staging is unreliable, especially for nodal staging, further work needs to be done to improve the radiologic identification of EMVI preoperatively. Systemic neoadjuvant chemotherapy, in patients with EMVI-positive and microsatellite stable tumours, may be beneficial given the known association of EMVI with metastatic disease and poorer prognosis. By considering the MMR and EMVI status of a tumour preoperatively, we can better tailor treatments specific to that patient. Our study lends further support to the growing evidence that EMVI confers a significantly worse prognosis in patients with colon cancer and should therefore carry greater weight in decision-making regarding neoadjuvant treatments to reduce the risk of systemic relapse and mortality. Further work needs to be undertaken to compare the relative efficacy of neoadjuvant versus adjuvant chemotherapy in right-sided cancers known to be EMVI-positive as some patients will fail to have adjuvant chemotherapy due to postoperative complications, thereby delaying recovery and missing the optimum window for treatment.
